# Microbial Metabolites: Critical Regulators in NAFLD

**DOI:** 10.3389/fmicb.2020.567654

**Published:** 2020-10-07

**Authors:** Xin Dai, Huiqin Hou, Wanru Zhang, Tianyu Liu, Yun Li, Sinan Wang, Bangmao Wang, Hailong Cao

**Affiliations:** ^1^Department of Gastroenterology and Hepatology, Tianjin Medical University General Hospital, Tianjin Institute of Digestive Diseases, Tianjin Key Laboratory of Digestive Diseases, Tianjin, China; ^2^Department of Pharmacy, General Hospital, Tianjin Medical University, Tianjin, China

**Keywords:** short-chain fatty acids, trimethylamine-N-oxide, bile acids, indole, fecal microbiota transplantation

## Abstract

Non-alcoholic fatty liver disease (NAFLD) is the most common form of chronic liver disease throughout the world. The relationship between gut microbiota and NAFLD has been extensively investigated. The gut microbiota is involved in the regulation of NAFLD by participating in the fermentation of indigestible food, interacting with the intestinal mucosal immune system, and influencing the intestinal barrier function, leading to signaling alteration. Meanwhile, the microbial metabolites not only affect the signal transduction pathway in the gut but also reach the liver far away from gut. In this review, we focus on the effects of certain key microbial metabolites such as short-chain fatty acids, trimethylamine-N-oxide, bile acids, and endogenous ethanol and indole in NAFLD, and also summarize several potential therapies targeting the gut–liver axis and modulation of gut microbiota metabolites including antibiotics, prebiotics, probiotics, bile acid regulation, and fecal microbiota transplantation. Understanding the complex interactions between microbial metabolites and NAFLD may provide crucial insight into the pathogenesis and treatment of NAFLD.

## Introduction

Non-alcoholic fatty liver disease (NAFLD) is one of the most common forms of chronic liver disease throughout the world. It is characterized by liver damage in the absence of excessive alcohol consumption. The spectrum of NAFLD extends from simple steatosis through non-alcoholic steatohepatitis (NASH) to cirrhosis and even hepatocellular carcinoma (HCC; Am and Day, 2017; Anstee et al., 2019). The global prevalence of NAFLD is estimated to be 25.24% nowadays and is still rising year by year. NAFLD not only causes severe hepatic injury but also is closely associated with type 2 diabetes, metabolic syndrome, hypertension, and cardiovascular disease. Lately, some experts even have reached consensus that metabolic dysfunction-associated fatty liver disease “MAFLD” is suggested as a more appropriate overarching term (Eslam et al., 2020). Thus, NAFLD is a major clinical and economic burden of the whole world (Younossi et al., 2018).

To date, the pathogenesis of NAFLD is not fully clarified. It is thought to be involved in complex interactions among diet, genetic susceptibility, and gut microbiota (Arab et al., 2017). Gut microbiota regulates the development and progression of NAFLD on the basis of the gut–liver axis. The concept of “gut–liver axis” was first proposed by Marshall in 1998, indicating close interaction between gut and liver. Gut-derived nutrients as well as other substances are absorbed and metabolized by enterocytes and reach the liver via the portal circulation. The slow blood flow in the liver sinusoids permits interactions between gut-derived signals and hepatocytes, other liver parenchymal cells, and liver immune cells; this is further promoted by the fenestrated endothelium in the sinusoids. Liver, the largest immune organ, has a remarkable capacity to recruit and activate immune cells in response to gut-derived metabolic or pathogen-derived signals (Scott, 2017; Albillos et al., 2020). The imbalance of gut–liver axis is increasingly recognized as a major factor in NAFLD.

Lately, the role of gut microbiota and microbial metabolites in NAFLD has attracted more attention. This review focuses on the effects of certain critical microbial metabolites in NAFLD and also summarizes several potential targets of gut–liver axis, gut microbiota, and its metabolites in NAFLD.

## Gut Microbiota and NAFLD

Several studies reveal the close relationship between gut microbiota and NAFLD in both human and mice. In human studies, NAFLD patients exhibited more gram-negative and fewer gram-positive bacteria compared with healthy volunteers. Besides, disease progression was correlated with phylum-level changes, such as an increase in *Proteobacteria* and a decrease in *Firmicutes* (Loomba et al., 2017). *Bacteroides* had a higher abundance in the stool and was independently associated with NASH, while proportions of *Prevotella* was lower in stool of NASH patients (Boursier et al., 2016). It was also reported that increased abundance of *Ruminococcus* was associated with fibrosis in NASH patients, as well as the abundance of *Streptococcus* (Nistal et al., 2019). Importantly, Da Silva et al. (2018) pointed that NAFLD was associated with dysbiosis independent of body mass index and insulin resistance.

Animal experiments further explain the causal links between dysbiosis and NAFLD as well as its possible mechanisms. Germ-free mice that received gut microbiota from mice with hyperglycemia and systemic inflammation, but not from healthy mice, developed significant hepatic steatosis (Le Roy et al., 2013). The germ-free mice were resistant to high-fat diet (HFD)-induced hepatic lipid accumulation compared with the conventionally fed mice (Rabot et al., 2010). These data indicated that the alteration of gut microbiota may play a causal role in the development of NAFLD, rather than a mere consequence of it.

How can gut microbiota regulate the development of NAFLD is the main concern of the researchers. Firstly, the gut microbiota actively participates in the fermentation of indigestible food such as carbohydrate, choline, and various kinds of proteins in the gut and facilitates the absorption of the metabolites into the portal vein and systemic circulation and thus regulate the energy balance (Jumpertz et al., 2011). Secondly, the gut microbiota and its metabolites interact with the intestinal mucosal immune system, to shape antigen recognition, recruitment, proliferation, and affect function. The gut microbiota can impact the liver, which is far away from where it actually resides through its metabolites effusing from the gut. The microbial metabolites can induce the systemic immune response and also the liver-specific immune response (Kau et al., 2011; Levy et al., 2017). Thirdly, the gut microbiota can influence the intestinal barrier function, such as the tight junction protein expression. Detrimental microbial metabolites destroy the gut barrier and rush into the portal vein and thus induce oxidative stress and hepatic steatosis (Manfredo Vieira et al., 2018). Last but not the least, the gut microbiota may lead to signaling alteration *via* microbial metabolites recognized by pattern recognition receptors. The microbial metabolites work as invisible hands that can reach the liver far away from where the gut microbiota actually resides and can regulate energy balance, immune response, intestinal barrier function, and signaling. In this way, microbial metabolites work as critical regulators in NAFLD.

## Microbial Metabolites and NAFLD

**FIGURE 1:**
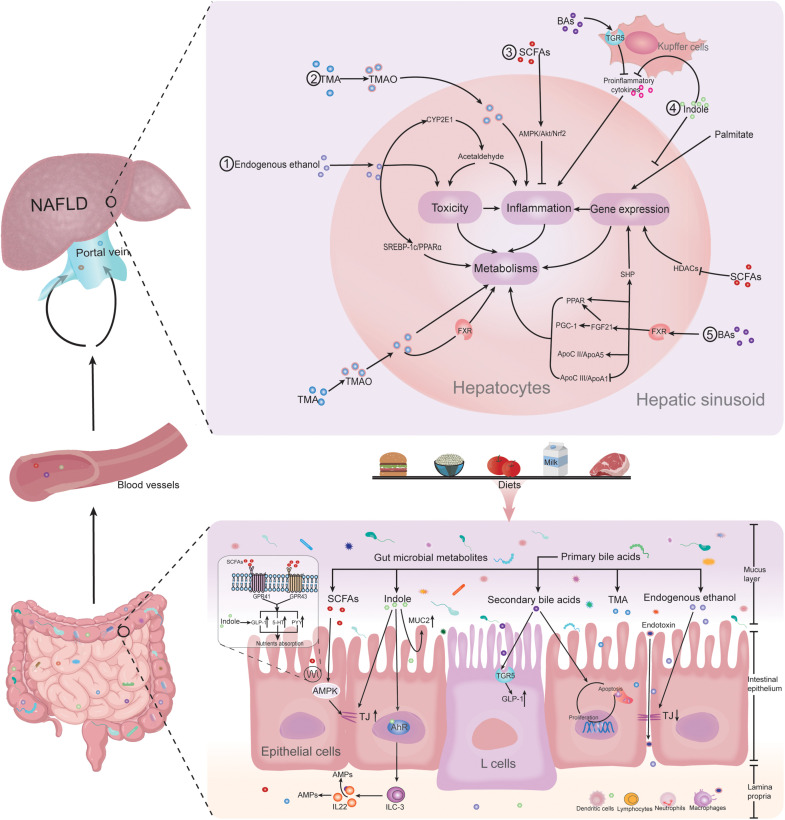
Gut microbiota-derived metabolites regulating NAFLD. Gut microbiota-derived metabolites of SCFAs, TMAO, bile acids, endogenous ethanol, and indole lead to the alteration of intestinal barrier function and the nutrition absorption in direct or indirect ways, as well as interact with the intestinal immune system. The microbial metabolites are then absorbed into the blood vessels and finally enter the liver through the portal vein. In the hepatic sinusoid, they are recognized as pattern recognition receptors or others, triggering downstream complex interaction between toxicity, inflammation, and gene expression responses, which will lead to metabolism alteration and ultimately regulate the progress of NAFLD. Abbreviations: NAFLD, non-alcoholic fatty liver disease; SCFAs, short-chain fatty acids; BAs, bile acids; TMA, trimethylamine; TMAO, trimethylamine N-oxide; MUC2, mucin 2; AMPK, AMP-activated protein kinase; TJs, tight junctions; AhR, aryl hydrocarbon receptor; TGR5, Takeda G protein-coupled receptor 5; GLP-1, glucagon-like peptide 1; ILC3, group 3 innate lymphoid cells; IL-22, interleukin-22; AMPs, antimicrobial peptides; HT-5, 5-hydroxytryptamine; PYY, peptide-YY; GPR41, G-protein receptor 41; GPR43, G-protein receptor 43; HDACs, histone deacetylases; SHP, small heterodimer partner; FXR, farnesoid X receptor; PPAR α, peroxisome proliferator-activated receptor α; PGC-1α, peroxisome proliferator-activated receptor γ coactivator-1α; FGF21, fibroblast growth factor 21; Apo CII, apolipoprotein CII; Apo A5, apolipoprotein A5; Apo CIII, apolipoprotein CIII; ApoA1, apolipoprotein A1; SREBP-1c, sterol regulatory element-binding protein-1c; CYP2E1, cytochrome P450 family 2 subfamily E member 1; and Nrf2, nuclear factor erythroid 2-related factor 2.

Gut microbiota-derived metabolites including short-chain fatty acids (SCFAs), trimethylamine-N-oxide (TMAO), bile acids (BAs), endogenous ethanol, indole, and other metabolites lead to the alteration of intestinal barrier function and the nutrition absorption in direct or indirect ways, as well as interact with the intestinal immune system. The microbial metabolites are then absorbed into the blood vessels and finally enter the liver through the portal vein. In the hepatic sinusoid, they are recognized as pattern recognition receptors or others, triggering downstream complex interaction between toxicity, inflammation, and gene expression responses, which will lead to metabolism alteration and ultimately regulate the progress of NAFLD (Figure 1).

### SCFAs and NAFLD

Short-chain fatty acids, mainly including acetate, propionate, and butyrate are the most abundant bacterial metabolites derived from intestinal bacterial fermentation of indigestible carbohydrate. As one of the important energy sources, SCFAs play a vital role in intestinal epithelial nutrition, energy metabolism, and physiological function regulation (Sanna et al., 2019). In addition, they also take part in physiological functional regulation as signal molecules. SCFAs activate the G-protein-coupled receptors (GPCRs) GPR41, GPR43, and GPR109A, which are expressed on adipocytes, hepatic cells, and colonic cells (Samuel et al., 2008; Maslowski et al., 2009; Singh et al., 2014).

The alleviation effects of SCFAs on NAFLD are well established in both human investigations and animal studies. In human investigations, comparing the composition of fecal microbiota of non-obese NAFLD patients and non-obese healthy subjects, SCFAs were apparently reduced in NAFLD group (Wang et al., 2016). Targeted propionate ester supplementation to colon significantly reduced intrahepatocellular lipid content in overweight adult humans (Chambers et al., 2015). In murine models, administration of sodium butyrate protected against Western-style diet-induced NASH, with significantly decreased hepatic steatosis and inflammation (Jin et al., 2015). Sodium acetate protected against nicotine-induced excess hepatic steatosis (Dangana et al., 2020). Interestingly, the cross-talk between SCFAs and NAFLD has also been found in early life stage. For example, maternal sucralose intake reduced SCFA-producing bacteria, depleted cecal butyrate production of offspring, and exacerbated hepatic steatosis in adulthood in mice (Dai et al., 2020).

There are several possible mechanisms by which SCFAs reduce the development of NAFLD. Firstly, SCFAs reduce fat accumulation in the adipose tissue. NAFLD is not a distinct and solitary condition; instead, it should be considered as the hepatic manifestation of metabolic syndrome, which includes central abdominal obesity along with other deteriorating metabolic disorders. Actually, visceral adipose tissue plays a critical role in obesity, insulin resistance, as well as NAFLD. The excessive accumulation of visceral fat increases the release of free fatty acids (FFAs) into the liver, which leads to an impaired ability of insulin to repress hepatic glucose production and also increases lipid synthesis (Ghosh et al., 2017). In parallel, FFA induces hepatic TNF expression through nuclear factor-kappa B (NF-κB) activation, which is considered as a key player in the development of NAFLD (Cordeiro et al., 2020). In addition, visceral adipose tissue secretes a variety of pro-inflammatory cytokines including leptin, resistin, and visfatin, as well as anti-inflammatory adipokines such as adiponectin at the same time. The pro-inflammatory and anti-inflammatory adipokines are in balance when visceral adipose tissue is in normal range. However, when adipose tissue overexpands, this balance tends to be lost, which induces systematic inflammation including the liver (Neuschwander-Tetri, 2017). Interestingly, SCFAs have a significant influence on visceral adipose tissue and fatty acid metabolism. For instance, SCFAs affect the transcriptional expression of adiponectin and resistin through modifying DNA methylation in obese mice (Yao et al., 2020). Acetate inhibits lipid accumulation by promoting lipolysis and fatty acid oxidation and inhibiting fatty acid synthesis in rabbits. Inhibited peroxisome proliferator-activated receptor α (PPAR α) and activated AMP-activated protein kinase (AMPK) and ERK1/2 signal pathways are related to the process in liver (Liu et al., 2019). Acetate is mainly responsible for the antilipolytic effects of SCFAs and acts via attenuation of hormone-sensitive lipase (HSL) phosphorylation in a Gi-coupled manner in human multipotent adipose tissue-derived stem (hMADS) adipocytes (Jocken et al., 2018).

Secondly, SCFAs can protect gut barrier. Butyrate enhances the intestinal barrier function by facilitating tight junction assembly *via* AMPK activation and attenuates HFD-induced steatohepatitis (Peng et al., 2009; Zhou et al., 2017). Furthermore, butyrate, acetate, and propionate activate intestinal nod-like receptor family pyrin domain containing 3 (NLRP3) inflammasome to stimulate the secretion of interleukin-18 (IL-18) and thus improve gut barrier integrity (Macia et al., 2015).

Thirdly, SCFAs regulate the intestinal mobility. Activation of GPR41 and GPR43 stimulates secretion of peptide-YY (PYY), glucagon-like peptide-1 (GLP-1), and 5-hydroxytryptamine (5-HT), which can inhibit gut motility, slow intestinal transit, as well as inhibit gastric emptying and food intake, thereby enhancing nutrient absorption, increasing energy harvest from the diet, and improving hepatic lipogenesis (Samuel et al., 2008; Tolhurst et al., 2012; Grasberger et al., 2013).

Fourthly, SCFAs directly enter the liver through the portal vein, where they contribute to suppressing inflammation and hepatic steatosis. Butyrate, acetate, and propionate induce AMPK activation, increase the expression of fatty acid oxidation gene, and suppress macrophage proinflammatory activation in the liver and, as a result, alleviate hepatic steatosis (Skelly et al., 2019). Stimulating HepG2 cells with sodium butyrate directly activates AMPK/Akt/Nrf2 signal pathway, which significantly reduces lipid deposition in hepatic cells (Endo et al., 2013). In addition, SCFAs appear to inhibit development of NAFLD at the epigenetic level. Aberrant histone modifications, especially histone acetylation, play a key role in NAFLD (Campisano et al., 2019). Histone deacetylases (HDACs) prevent gene transcription by removing histone-bound acetyl groups. Butyrate and, to some level, propionate and acetate are HDAC inhibitors and regulate gene expression through modulation of chromatin state (Kolodziejczyk et al., 2019). For instance, deletion of sirtuin-3 (SIRT3), one of the most studied member of the family Class III HDACs, promotes HFD-induced NAFLD, and sodium butyrate supplementation can reduce such liver damage (Chen et al., 2019).

### TMAO and NAFLD

Choline from meat, yolk, and dairy products can be metabolized to trimethylamine (TMA) by gut microbiota. TMA is oxidized by hepatic flavin monooxygenases and forms TMAO in the liver subsequently and then releases into circulation and finally is eliminated in kidney (Janeiro et al., 2018). Compared with mice fed on control diet, supplementation with choline in the diet of conventional mice increases the plasma levels of TMAO. However, choline supplementation does not increase the levels of TMA (of course TMAO) in germ-free mice. It indicates that TMAO is only derived from gut microbial metabolism (Zhu et al., 2016).

Most studies reveal that TMAO plays a negative role in the development of atherosclerosis, obesity, as well as NAFLD (Wang Z. et al., 2011; Koeth et al., 2013; Schugar et al., 2017). In human studies, an increasing body of evidence showed that TMAO levels were significantly associated with NAFLD. A large sample of a hospital-based case–control study and a community-based cross-sectional study in China presented positive associations of the circulating TMAO levels and two of its nutrient precursors including choline and betaine, with the presence and severity of NAFLD (Chen et al., 2016). In a cross-sectional investigation in Italy, the researchers found that TMAO levels increased along with BMI and were positively associated with fatty liver index (FLI), a predictor of NAFLD (Barrea et al., 2018). They also showed that vitamin D deficiency and high circulating TMAO levels were associated with the severity of NAFLD (Barrea et al., 2019). A cross-sectional study in a male Mediterranean population revealed that several biogenic amine levels in urine, including TMAO in patients with metabolic syndrome, were higher compared with their counterparts without metabolic syndrome (Ntzouvani et al., 2017). Considering the critical participation of TMAO in the progression of NAFLD, some researchers suggested that it could be used as a “liquid biopsy” in the predictive diagnosis of NASH (Aragonès et al., 2020).

In animal experiments, substantial data show that TMAO increased hepatic triglyceride accumulation and impaired liver function (Tan et al., 2019). Besides, the potential mechanism that show that TMAO aggravates NAFLD is also investigated. It may lie in the fact that TMAO increases insulin resistance and induces glucose metabolism disorder. The expressions of hepatic insulin signaling-related genes decrease dramatically in the mice fed with TMAO (Gao et al., 2014). Besides, TMAO aggravates inflammation in adipose tissue (Koeth et al., 2013; Gao et al., 2014). Importantly, a variety of pro-inflammatory molecules are secreted by adipose tissue. This condition leads to macrophage activation and then induces the liver to produce C-reactive protein (CRP) and initiates a pro-inflammatory signaling pathway. Nowadays, adipose tissue-derived macrophage is considered as a key player in the development of NASH (Cordeiro et al., 2020). In parallel, TMAO also reduces the conversion of cholesterol into BAs, thus affecting lipid absorption and cholesterol homeostasis (Koeth et al., 2013). In addition, TMAO increases BA synthesis and shifts hepatic BA composition mediated by hepatic farnesoid X receptor (FXR) signaling (Tan et al., 2019).

However, some studies suggest the opposite view. Zhao et al. (2019b) showed that oral TMAO intervention inhibited intestinal cholesterol absorption, ameliorated hepatic endoplasmic reticulum stress, and reduced cell death under cholesterol overload, thereby attenuating high-fat, high-cholesterol diet-induced steatohepatitis in mice. More studies are needed to evaluate the influence of TMAO on NAFLD, and the potential mechanism should be discussed in the future.

### BAs and NAFLD

Primary BAs are synthesized in the liver, stored in the gallbladder, and then released into the duodenum. In the gut, they are converted into secondary BAs via gut microbiota. BAs are confirmed to be closely related to expression of tight junction proteins as well as the proliferation and apoptosis of intestinal epithelial cells (Camilleri, 2019). Most of the BAs are reabsorbed into the portal vein and recycled by the liver, which is named enterohepatic circulation. However, some BAs remain in the blood and act as signaling molecules (Liu et al., 2020). BAs not only play an important role in maintaining hepatic glucose, cholesterol, and triglyceride homeostasis but also act as signaling molecules via Takeda G protein-coupled receptor 5 (TGR5), FXR, and other receptors (Jiao et al., 2018). Both abundant clinical trials and animal experiments have confirmed that BAs are essential to the development of NAFLD (Rao et al., 2016; Arab et al., 2017; Chávez-Talavera et al., 2017). Puri et al. (2018) analyzed the plasma BA profile in biopsy-proven NAFL, NASH, and healthy controls and found that altered circulating BA composition was associated with NAFLD and, most importantly, correlated with histology of NASH, which indicated that BAs may participate in the development from NAFL to NASH.

As BAs are the essential ligands of FXR and TGR5, BAs regulate the hepatic steatosis by multiple mechanisms *via* FXR and TGR5 activation (Chiang and Ferrell, 2020). (1) Inhibiting lipogenesis. Deficiency of *Fxr* in mice increases hepatic and serum triglycerides and promotes atherosis, suggesting that FXR regulates lipid and lipoprotein metabolism (Sinal et al., 2000). Activation of FXR induces apolipoprotein CII (Apo CII) and apolipoprotein A5 (Apo A5) but inhibits apolipoprotein A1 (ApoA1) and apolipoprotein CIII (Apo CIII), ultimately activating lipoprotein lipase in very low-density lipoprotein (VLDL) particles to reduce serum triglycerides (Claudel et al., 2003). Besides, FXR activation induces PPAR α expression, which stimulates fatty acid β-oxidation, and thus reduces lipid accumulation (Pineda Torra et al., 2003). Furthermore, FXR downstream fibroblast growth factor 21 (FGF21) can induce PPAR α and peroxisome proliferator-activated receptor γ coactivator-1α (PGC-1α) to stimulate fatty acid oxidation and energy metabolism (Schlein et al., 2016). (2) Regulating glucose metabolism. In the gut, TGR5 activation stimulates GLP-1 secretion from intestinal enteroendocrine L cells to increase insulin secretion and improve glucose tolerance (Thomas et al., 2009). Besides, FXR activation inhibits the expression of gluconeogenic genes by inducing small heterodimer partner (SHP), which reduces serum glucose in wild-type mice. SHP also inhibits growth hormone-mediated induction of gluconeogenesis by inhibiting signal transducer and activator of transcription 5 (STAT5) transactivation (Kim et al., 2012). (3) TGR5 serves as a negative modulator of NF-κB-mediated inflammation. Activation of TGR5 significantly inhibits the mRNA levels of inducible nitric oxide synthase (iNOS), monocyte chemoattractant protein-1 (MCP-1), cyclooxygenase-2 (COX-2), interleukin-6 (IL-6), and other pro-inflammatory factors, thus alleviating the development of NASH (Wang Y. D. et al., 2011). Besides, BAs and TGR5 activation block NLRP3 inflammasome-dependent inflammation, which is considered to participate in NAFLD (Guo et al., 2016; Zilu et al., 2019). (4) Interestingly, activation of FXR has been shown to induce Tgr5 gene transcription via a FXR binding site located in the Tgr5 gene promoter. In this way, FXR and TGR5 signal form a cross-talk to regulate the hepatic steatosis closely (Hu et al., 2020).

### Endogenous Ethanol and NAFLD

Dietary carbohydrate can be fermented by gut microbiota into ethanol, which then enters the bloodstream and finally eliminated through the liver. It is reported that the ethanol-producing bacteria mainly include *Bacteroides fragilis*, *Escherichia*, *Bifidobacterium adolescentis*, and *Clostridium thermocellum* (Amaretti et al., 2007). Both animal studies and clinical trials indicate that gastrointestinal ethanol levels significantly increase in NAFLD compared with the controls, and the ethanol levels are associated with gut microbiota (Cope et al., 2000; Zhu et al., 2013). For instance, Michail et al. (2015) pointed that children with NAFLD had a significantly higher level of ethanol compared with the controls, which were associated with a greater abundance of *Gammaproteobacteria* and *Prevotella.* However, Engstler et al. (2016) demonstrated in mice models and patients that the increased blood ethanol levels in NAFLD might result from insulin-dependent impairments of ethanol dehydrogenase activity in the liver rather than an increase in endogenous ethanol synthesis. Remarkably, they emphasized that they just evaluated NAFLD in early stage. It may suggest that future studies are required to uncover the exact effects of endogenous ethanol on NAFLD at different stage.

Increasing studies explore the potential mechanisms between endogenous ethanol and NAFLD. On one hand, in the liver, ethanol exposure can induce the lipid deposition and inhibit fatty acid β-oxidation by regulating sterol regulatory element-binding proteins-1c (SREBP-1c) and PPAR α, which are important factors involved in lipid metabolism directly and indirectly, thus exacerbating hepatic steatosis (Zhang et al., 2018). In addition, ethanol exacerbates hepatic inflammation and fibrosis. Ethanol can increase the activity of cytochrome P450 family 2 subfamily E polypeptide 1 (CYP2E1) to catalyze ethanol oxidation and produce acetaldehyde, peroxide, and free radicals that might cause inflammatory cascade reactions in the liver. CYP2E1 is also a potent profibrotic signal. The fact that patients suffering from NASH have significantly increased CYP2E1 levels strongly supports this point (Zong et al., 2012). On the other hand, in the gut, ethanol and its metabolites, especially acetaldehyde, appear to disturb the tight junctions and increase the intestinal permeability, thereby destroying the gut barrier functions. The endotoxin and the other detrimental microbial metabolites may rush into the portal vein through the impaired gut barrier and upregulate pro-inflammatory cytokine production in the liver, which finally exacerbates the development of NAFLD (Llorente and Schnabl, 2015).

### Indole and NAFLD

Dietary tryptophan can be metabolized by gut microbiota into indole and its derivatives, which mainly include indole-3-acetic acid (IAA), indole-3-propionic acid (IPA), indole-3-lactic acid, indole-3-carboxylic acid, and tryptamine (Canfora et al., 2019). Recently, a growing body of evidence supports the idea that indole and its derivatives exert a protective role in NAFLD. At the cellular levels, IAA mitigates palmitate-induced lipogenic gene expression in hepatocytes and suppresses pro-inflammatory cytokine production in RAW264.7 macrophages (Krishnan et al., 2019). At the animal levels, IAA significantly attenuates HFD-induced hepatic steatosis in male C57BL/6 mice. The insulin resistance, dyslipidemia, oxidative, and inflammatory stress are also ameliorated (Ji et al., 2019). The levels of IAA in both liver and cecum of HFD-fed mice are decreased relative to those in control mice (Krishnan et al., 2019). At the human levels, the circulating levels of indole in obese subjects are significantly lower than that in lean subjects and, interestingly, accompanied by increased liver fat content (Ma et al., 2020).

Besides IAA, the protective effects of other indole derivatives on NAFLD have also been studied. Chung et al. (2015) found that indole derivative NecroX-7 improved hepatic steatosis and fibrosis through suppression of whole-cell ROS/RNS and inflammatory responses in murine NASH models. Zhao et al. (2019a) reported that administration of IPA also attenuated NASH by inhibiting the production of endotoxin in the gut and improving the expression of tight junction proteins. Konopelski et al. (2019) showed that IPA obviously reduced weight gain in a mice experiment. However, the researchers did not evaluate the change of liver. Although the studies about IPA and NAFLD are not so abundant, considering that IPA has the effects of reducing oxidative stress and improving glucose metabolism (Abildgaard et al., 2018), we are still looking forward that IPA can be a candidate for treatment of NAFLD in the future.

The mechanism of how indole and its derivatives reduce hepatic steatosis is still not fully illuminated. In the gut, indole and its derivatives have been described as activators of the aryl hydrocarbon receptor (AhR). AhR-mediated signaling in group 3 innate lymphoid cells (ILC3) promotes interleukin-22 (IL-22) production, which improves mucosal defense *via* the induction of antimicrobial proteins (AMPs; Gronke et al., 2019). Indole stimulates mucin production and enhances tight junction proteins, thus protecting the gut barrier function. Also, indole induces the release of the GLP-1, which stimulates insulin secretion, suppresses appetite, and inhibits gastrointestinal motility (Taleb, 2019). In addition, as indole treatment stimulates the expression of 6-phosphofructo-2-kinase/fructose-2,6-biphosphatase 3 (PFKFB3), a master regulatory gene of glycolysis, and suppresses macrophage proinflammatory activation in a PFKFB3-dependent manner, and, moreover, myeloid cell-specific PFKFB3 disruption exacerbates the severity of HFD-induced hepatic steatosis and inflammation, researchers speculate that the protective role of indole in NAFLD is in a myeloid cell PFKFB3-dependnet manner (Ma et al., 2020).

### Other Metabolites and NAFLD

Other gut microbial metabolites such as phenylacetate, succinate, N,N,N-trimethyl-5-aminovaleric acid (TMAVA), 3-(4-hydroxyphenyl) lactate, and ketones also take part in NAFLD. Their effects potentially work in different ways—exacerbating, alleviating, or sometimes controversial.

Phenylacetate is an important gut bacterial metabolite derived from phenylalanine. The levels of phenylacetate are up-regulated in the blood of non-diabetic obese women with NASH. In addition, chronic administration of phenylacetate triggers hepatic steatosis in mice (Hoyles et al., 2018).

Succinate is also derived from intestinal bacterial fermentation of indigestible carbohydrate (Canfora et al., 2019). Animal studies suggest that succinate plays an important role in the prevention and treatment of obesity and insulin resistance by activating intestinal gluconeogenesis (Vadder et al., 2016). However, controversial data are also increasing. Serena et al. (2018) reported that increased circulating concentrations of succinate and increased abundance of succinate-producing bacteria were associated with obesity and abnormal glucose metabolism in human. Tannahill et al. (2013) showed that succinate was an inflammatory signal that induces interleukin-1β (IL-1β) through hypoxia-inducible factor 1α (HIF-1α), therefore contributing to progression of insulin resistance. It seems that more investigations are needed to uncover the exact relationship between succinate and NAFLD.

Trimethyl-5-aminovaleric acid is another novel gut bacterial metabolite derived from trimethyllysine. Significantly elevated serum levels of TMAVA are detected in patients with liver steatosis. Mechanically, TMAVA may inhibit γ-butyrobetaine hydroxylase, which reduces carnitine synthesis and suppresses mitochondrial hepatic fatty acid oxidation in the liver to exacerbate hepatic steatosis (Zhao et al., 2020).

## The Microbial Metabolites as a Potential Therapeutic Target in NAFLD

As the microbial metabolites serve as invisible hands connecting the gut microbiota and NAFLD via the gut–liver axis, therapeutic approaches targeting the gut–liver axis, and modulation of gut microbiota and its metabolites could be promising.

### Antibiotics

Theoretically, antibiotics can reduce the gut bacterial overgrowth and diminish the translocation of microbial metabolites, resulting in therapeutic effects on NAFLD. Antibiotics efficacy of treating NAFLD has been demonstrated in various studies. Several clinical investigations indicated that short-term administration of rifaximin appeared to significantly improve NAFLD with reduced circulating endotoxins and serum transaminases (Gangarapu et al., 2015; Abdel-Razik et al., 2018). However, one clinical trial presented opposite results. In an open-label pilot study reported by Cobbold JFL, rifaximin showed little therapeutic effects against hepatic lipid content and insulin sensitivity (Cobbold et al., 2018). On one hand, the inconsistency may be due to the small sample size, the relatively low treatment dose, or the short duration of the clinical study. On the other hand, this may be because rifaximin, as a broad-spectrum antibiotic, can affect not only the harmful bacteria but also the beneficial ones due to their wide range of action. Future therapies targeting the gut microbiota will need to be more nuanced to result in beneficial metabolic and inflammatory modulation.

Besides rifaximin, a number of other antibiotics have been reported for the treatment of NAFLD in mice, such as cidomycin (Wu et al., 2008), polymyxin B, and neomycin (Bergheim et al., 2008). However, the evidence is limited to animal studies. More clinical researches are needed in the future.

There are several factors that limit the use of antibiotics in the treatment of NAFLD. Firstly, some antibiotics may be associated with adverse metabolic effects. For example, a reduction in the ratio of secondary to primary BAs was observed after rifaximin administration (Kakiyama et al., 2008). Secondly, long-term use of antibiotics should be cautious due to the increasing possible side effects. Thirdly, antibiotics may select for antibiotic-resistant strains in the human gut, resulting in less effective treatment. In addition, the use of antibiotics in immunocompromised patients may increase the risk of infective endocarditis and bacteremia, which should be with great caution. Collectively, the administration of antibiotics seems to alleviate NAFLD, but the clinical use is still questionable.

### Prebiotics

Oral supplementation of SCFAs such as butyrate is challenging because of its rancid smell and unpleasant taste (Chambers et al., 2018; Chen et al., 2020). Butyrate enema may promote visceral hypersensitivity (Long et al., 2018). Several randomized crossover trials show that colonic infusions of SCFA mixtures increase fat oxidation, energy expenditure, and PYY, and decrease lipolysis in overweight and obese men. However, the study objects are so limited (van der Beek et al., 2016; Long et al., 2018). As direct SCFA supplementation is hardly accepted, quite a number of researchers focus on prebiotics that can yield SCFAs for NAFLD treatment. Prebiotics are food ingredients that stimulate the growth and activity of beneficial bacteria. They can also be fermented by gut bacteria to beneficial metabolites such as SCFAs. In a clinical study, inulin, an important kind of prebiotics, was shown to decrease hepatic lipogenesis and plasma triglyceride level (Letexier et al., 2003). Another double-blind, placebo-controlled trial also showed that metronidazole with inulin supplementation can reduce ALT beyond that achieved after very-low-calorie diet in patients with NAFLD (Chong et al., 2020). However, generally speaking, the results are inconsistent. Some show only minimal or no effect (Dewulf et al., 2013; Vulevic et al., 2013; Canfora and Blaak, 2015; Canfora et al., 2017). Moreover, high-quality clinical evidence appears insufficient. On one hand, their various effects may relate to the characteristics of the specific fibers, including their fermentation type, site of fermentation, amount, and type of metabolites produced. On the other hand, the consumption of prebiotics in excess of 30 g/day would also cause adverse gastrointestinal effects such as abdominal distension (Kleessen et al., 1997). In addition, some important clinical assessment indicators, like liver biopsy, are difficult to carry out widely. In the future, more strict large-scale clinical studies are needed to achieve a comprehensive understanding of the potential for translating the positive effects of prebiotics, particularly in animal to human clinical application.

### Probiotics

Probiotics are a collection of live bacteria with beneficial effects on host. Until now, a large amount of clinical investigations with different kinds and preparations of probiotics have been reported to treat NAFLD (Aller et al., 2011; Miccheli et al., 2015; Sepideh et al., 2016; Manzhalii et al., 2017). In a double-blind, placebo-controlled pilot study, the levels of alanine aminotransferase and liver ultrasound brightness were improved after short-term probiotic *Lactobacillus rhamnosus* strain GG administration in patients with NAFLD (Vajro et al., 2011). VSL#3 is a high-concentration probiotic preparation of eight live freeze-dried bacterial species (Panetta et al., 2020). Alisi et al. (2014) treated obese children with NAFLD with VSL#3 and found that pediatric NAFLD improved. Kobyliak et al. (2018) showed that the combination of multi-strain probiotics reduced liver fat and inflammation levels in NAFLD patients. Similarly, a probiotic capsule containing *Lactobacillus plantarum*, *Lactobacillus delbrueckii*, *Lactobacillus acidophilus*, *Lactobacillus rhamnosus*, and *Bifidobacterium bifidum* reduced liver fat and AST level in patients with NASH (Wong et al., 2013). Collectively, there is increasing evidence supporting the idea that probiotics can be used as potential therapeutics for NAFLD. However, the effect varies due to bacterial strains, colonization, preservation, and quantity. It is also associated with treatment time. The combined administration of multiple probiotic strains may be more effective than a single one. Besides, until now, all the clinical trials are limited by a small sample size and uniformity between participants. More strict randomized controlled trials are needed in the future.

### BA Regulation

Obeticholic acid (OCA) is a synthetically modified analog of chenodeoxycholic acid and acts as a potent agonist of FXR. In a phase 3 clinical trial, OCA has yielded encouraging results that it can improve NASH with no worsening of fibrosis or fibrosis improvement. However, the effects of OCA on the co-primary endpoint of NASH resolution are not achieved. The side effects including pruritus and elevated low-density lipoprotein (LDL) cholesterol levels remain a concern. The safety and efficiency of OCA will be assessed in real-world populations in the long term (Ray, 2014; Eslam et al., 2019; Younossi et al., 2019; Siddiqui et al., 2020).

Other FXR agonists such as GS-9674 and LJN452 are both in the phase 2 clinical trial for NASH. The BA derivative sodium salt INT-767 is a FXR and TGR5 dual agonist. INT-767 is a more powerful FXR agonist than OCA and is in a phase 1 clinical trial for NASH. In addition, FGF19 and FGF21 analogs are also promising therapeutic targets for NAFLD. NGM282, an engineered FGF19 analog, rapidly and significantly reduces liver fat content in patients with biopsy-confirmed NASH in a phase 2 trial (Harrison et al., 2018, 2020). BMS-986036 is a PEGylated FGF21 analog. In a phase 2a trial, BMS-986036 has shown promising improvements in several NASH-related outcomes. However, long-term benefits on more essential outcomes such as liver histology, cirrhosis development, and survival are required for further verification (Verzijl et al., 2020).

### Fecal Microbiota Transplantation

Several studies investigate fecal microbiota transplantation (FMT) impacts on the gut microbial metabolites related to NAFLD. A double-blind randomized controlled pilot study showed that single lean vegan-donor FMT in patients with metabolic syndrome resulted in detectable changes in gut microbiota composition but no change in TMAO production capacity (Smits et al., 2018). According to another report, a stool transplant, which was either autologous or from lean healthy volunteers to the patients with metabolic syndrome, showed a significant rise in fecal butyrate level (Aron-Wisnewsky et al., 2019). Until now, there are five clinical trials evaluating the FMT impacts on NAFLD (Philips et al., 2017; Ebrahimzadeh Leylabadlo et al., 2020). It has not been reported that FMT raises severe adverse events in NAFLD in published results. However, they are just preliminary results. More large cohorts of patients including different etiologies and at different stages of NAFLD should be evaluated. There are several factors that affect the clinical efficacy of FMT, for instance, the route of delivery, the amount of bacteria, the frequency of transplantation, bowel preparation before transplantation, and diet after transplantation. More investigations will be needed in the future.

## Conclusion and Perspective

Non-alcoholic fatty liver disease is the most common form of chronic liver disease throughout the world. The relationship between gut microbiota and NAFLD has been extensively investigated. Microbial metabolites, such as SCFAs, TMAO, BAs, endogenous ethanol, and indole work as critical regulators in NAFLD. Although alterations in microbial metabolites in NAFLD are definite, conclusions from various studies are inconsistent because of unified research standards, different determination methods, and complicated signaling pathways. The novel techniques targeting gut microbial metabolites are emerging, and some of them are awaiting approval for NASH. However, confirmed clinical trials are still not enough, and more carefully designed, larger-scale clinical studies will still be needed in the future.

## Author Contributions

XD, HH, and WZ performed the literature search and drafted the manuscript. TL, YL, and SW provided critical intellectual contributions. HC and BW directed the research and made the critical revision. All authors have read and approved the final version of the manuscript.

## Conflict of Interest

The authors declare that the research was conducted in the absence of any commercial or financial relationships that could be construed as a potential conflict of interest.
